# The potential effect modifying role of nutrition, physical activity, and body mass index on the association between air pollution and adverse birth and early-life health outcomes: a scoping review

**DOI:** 10.1088/2515-7620/adc903

**Published:** 2025-04-16

**Authors:** Christian Sewor, Kristen M Rappazzo, Maggie L Clark

**Affiliations:** 1 Department of Environmental and Radiological Health Sciences, Colorado State University, Fort Collins, CO, United States of America; 2 U.S. Environmental Protection Agency, Office of Research and Development, Center for Public Health and Environmental Assessment, Research Triangle Park, Durham, NC, United States of America

**Keywords:** effect modification, air pollution, birth outcomes, maternal nutrition, childhood BMI, physical activity

## Abstract

**Background.:**

Air pollution is a prominent contributor to the burden of adverse birth and early child health outcomes. However, considerable heterogeneity of impacts has been observed, which may be due to limited exploration of key effect modifiers. This scoping review was conducted to synthesize evidence on the potential effect modifying roles of nutrition, physical activity, and body mass index (BMI) on the associations between early-life air pollution exposures and adverse birth and early-life health outcomes.

**Methods.:**

PubMed, Web of Science, and Scopus databases were systematically searched for relevant studies through July 2023. Studies were included if they were conducted amongst pregnant women or individuals between 0–17 years, provided empirical evidence on associations between air pollution exposure and adverse birth and/or early-childhood health outcomes, and conducted effect modification-related analyses by maternal (i.e., in-utero) or early childhood nutrition, physical activity, or BMI. Data from selected studies were abstracted and summarized based on study design, population characteristics, and the exposures, outcomes, and effect modifiers assessed.

**Results.:**

A total of 13 studies were included; 10 were cohort studies, and 3 were cross-sectional studies. All but one of the studies explored the impact of ambient air pollutants (particulate matter, nitrogen dioxide, ozone, ultra-fine particles, elemental carbon, and black carbon) prenatally or in early life on adverse birth (preterm birth, birth weight, low birth weight) and early childhood outcomes (childhood obesity). Effect modifiers examined included pre-pregnancy BMI (n = 5 studies), maternal and child dietary characteristics (n = 7 studies), and child physical activity patterns (n = 2 studies).

**Discussion.:**

Evidence for effect modification, although present, was inconsistent and weak. Consideration should be given to exploring effect modification of air pollution-related impacts to help explain heterogeneity of associations observed across populations, a key knowledge gap limiting public health messaging strategies.

## Graphical Abstract

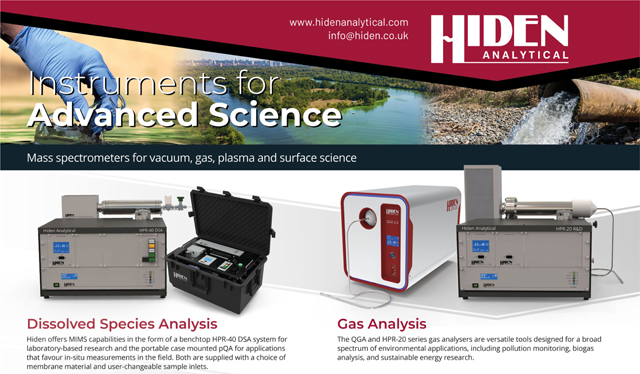

## 1. Introduction

Air pollution, which includes indoor and ambient sources resulting from various anthropogenic activities, is a prominent contributor to the global health burden. In 2019, 99% of the world’s population was reported to live in areas where the World Health Organization’s air quality guideline levels were not met [1]. Air pollution in that same year was estimated to contribute to 6.67 million deaths globally [2]. The deleterious effect of air pollution is often high among risk groups such as pregnant women and children. For instance, air pollution is attributed to 30% of lower respiratory infections as well as 20% of infant mortality in the first month of life [2]. In 2019, total PM_2.5_ (particulate matter less than 2.5 microns in diameter) exposure resulting from household and ambient air pollution was attributed to 15.6% and 35.7% of all globally occurring low birth weight (LBW) and preterm birth (PTB) rates, respectively [3].

Pooled estimates from a systematic review by Ghosh *et al* [3] reported a 10 μg m^−3^ increment in ambient PM_2.5_ was associated with decreased birth weight as well as increased risks of LBW and PTB, respectively. Similar systematic reviews and meta-analyses by Ju *et al* [4] and Nyadanu *et al* [5] have reported that air pollutants such as particulate matter (PM_2.5_, PM_10_), sulfur dioxide (SO_2_), and ozone (O_3_) were associated with reductions in birth weight and an increased risk of PTB, LBW, and spontaneous abortion. Also, meta-analyses by Luo *et al* [6] and Amegah *et al* [7], which focused on the impact of cooking with polluting fuels, which are major contributors to household air pollution, found them to be associated with increased odds of LBW, stillbirth, and reduction in birth weight. Air pollution exposure has also been linked to an increased risk of early child health outcomes such as childhood obesity. For instance, systematic reviews by Parasin *et al* [8] and Huang *et al* [9] noted that PM_1,_ PM_2.5_, PM_10_, and nitrogen dioxide (NO_2_) exposure were independently associated with an increased risk of childhood obesity. It is, however, worth noting that while evidence for air pollution and adverse birth and early child health outcomes is strong, high heterogeneity of associations is reported in these reviews [4, 5, 8, 9]. In some cases, the heterogeneity of associations remains even among similar study designs, which raises concerns about whether other relevant factors may be affecting this complex association. For instance, reviews have reported differences in the relationships between air pollution and health outcomes with respect to various socioeconomic and lifestyle factors [10–12].

Along with the potential variations in the relationships between air pollution and health outcomes by socioeconomic and lifestyle factors in early life, maternal nutrition plays a substantial role in ensuring good fetal health and minimizing the risk of adverse birth outcomes. As noted by Chia *et al* [13], healthy dietary patterns, characterized by high intakes of vegetables, fruits, whole grains, low-fat dairy, and lean protein foods, were associated with a reduced risk of PTB and higher birth weight, whereas unhealthy dietary patterns, characterized by high intakes of refined grains, processed meat, and foods high in saturated fat or sugar, were associated with increased risks of these outcomes. Similarly, there is also a growing body of evidence on the impact of characteristics such as physical activity patterns, pre-pregnancy body mass index (BMI), and alcohol intake on adverse birth and early childhood health outcomes. Evidence from reviews indicates that increased physical activity [14, 15], healthy pre-pregnancy BMI [16, 17], and low alcohol intake [18] are associated with reduced risk of adverse birth (e.g., PTB, LBW) and early childhood outcomes (e.g., childhood obesity).

Given the protective role of good nutrition, increased physical activity, and healthy BMI levels, there is a growing interest in how these factors may ameliorate the effect of air pollution on adverse birth and early childhood outcomes [19–21]. Air pollution leads to poor outcomes by inciting a cascade of pathologic mechanisms such as oxidative stress and systemic inflammation. Healthy nutrition, physical activity practices, and healthy levels of BMI, particularly before and during pregnancy as well as during early life, may alleviate the negative impact of air pollution on fetal and early child health by blocking or reducing these pathways [22–25].

**Figure 1.**
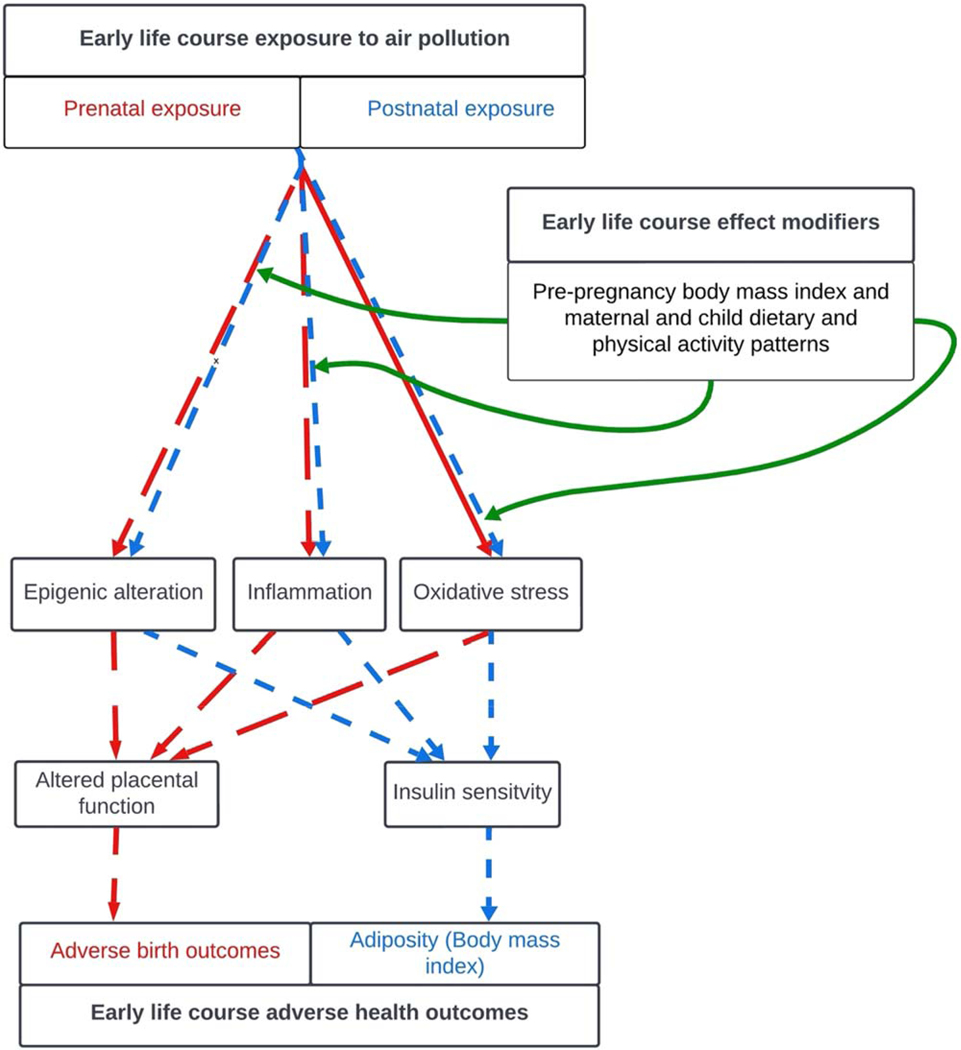
Hypothesized impact of effect modifiers on the relationship between prenatal and postnatal air pollution and adverse birth and early childhood health outcomes.

Summative evidence that formally synthesizes how epidemiological studies have evaluated the effect modifying role of these characteristics is needed. A scoping review can be a useful tool (1) when the literature surrounding a particular research question has not been comprehensively reviewed, and (2) to conceptualize boundaries of a topic that exhibits a heterogeneous nature not amenable to a more precise review [26]. Therefore, to meet and further the state-of-the-science, we have chosen to include a wide range of early-life outcomes relevant to this research question for potential inclusion in our study. Our motivation is to summarize the evidence and identify gaps to inform future research aimed at garnering a better understanding of the common biological pathways (i.e., oxidative stress and systemic inflammation) that underlie mechanisms due to air pollution exposure during pregnancy and early life by focusing on the potential effect modifying role of factors that promote antioxidative or anti-inflammatory conditions; mechanistic processes that are hypothesized to contribute to many air pollution-related birth and early childhood health outcomes (figure 1). Our scoping review was thus conducted to synthesize the body of evidence on the effect modifying role of nutrition, physical activity, and BMI on the association between air pollution and adverse birth and early child health outcomes.

## 2. Method

This scoping review followed the prescribed PRISMA guidelines for scoping reviews [27].

### 2.1. Eligibility criteria

#### 2.1.1. Inclusion criteria

Articles were considered for inclusion if they had satisfied the following: (a) were peer-reviewed published primary studies employing cross-sectional, case-control, cohort, or randomized controlled trial study designs or variants, (b) were conducted in a human population (if they were either conducted amongst pregnant women or amongst individuals between 0–17 years), (c) provided empirical evidence on the association between air pollution exposure or its related effect from cooking emissions (household/indoor and ambient) and the risk of any of the listed outcomes in the search statement (see below) and results on potential effect modification. Effect modifiers considered were maternal (before or during pregnancy) and/or early childhood nutrition, pre-pregnancy BMI, or physical activity with any assessment method.

#### 2.1.2. Exclusion criteria

Studies that failed to meet any of the inclusion criteria were excluded. Also, studies conducted amongst individuals with any chronic disease were excluded. Studies were excluded if they were published in any language besides English and if they were reviews. Selected articles were retrieved in full and further assessed for eligibility. The reference lists of all included studies and the related reviews were examined to identify additional eligible studies.

### 2.2. Information sources

PubMed, Web of Science, and Scopus databases were systematically searched from inception to the end of July 2023.

### 2.3. Search

The search statement used in both the PubMed and Web of Science databases was as follows: ((‘Household Air Pollution’ OR ‘Indoor air pollution’ OR ‘Air pollution’ OR ‘Ambient air pollution’) AND (Nutrition OR ‘Maternal Nutrition’ OR Food OR Diet OR Nutrient OR ‘prenatal body mass index’ OR ‘pre-pregnancy body mass index’ OR ‘body mass index’ OR ‘BMI’ OR ‘physical activity’) AND (‘Low birthweight’ OR LBW OR ‘Preterm birth’ OR PTB OR ‘Fetal death’ OR ‘Small for gestational age’ OR ‘Intrauterine growth restriction’ OR ‘Birth outcome’ OR ‘childhood obesity’ OR ‘Infant pneumonia’ OR ‘Lower Respiratory Infection’ OR ‘Adverse birth outcome’ OR ‘Birthweight’)).

The search statement used in the Scopus database was as follows: ((TITLE-ABS(‘Household Air Pollution’ OR ‘Indoor air pollution’ OR ‘Air pollution’ OR ‘Ambient air pollution’)) AND (TITLE-ABS(nutrition OR ‘Maternal Nutrition’ OR food OR diet OR nutrient OR ‘prenatal body mass index’ OR ‘pre-pregnancy body mass index’ OR ‘body mass index’ OR ‘BMI’ OR ‘physical activity’)) AND (TITLE-ABS(‘Low birthweight’ OR LBW OR ‘Preterm birth’ OR PTB OR ‘Fetal death’ OR ‘Small for gestational age’ OR ‘Intrauterine growth restriction’ OR ‘Birth outcome’ OR ‘childhood obesity’ OR ‘Infant pneumonia’ OR ‘Lower Respiratory Infection’ OR ‘Adverse birth outcome’ OR ‘Birthweight’)).

### 2.4. Selection of sources of evidence

The initial selection involved identifying and screening the articles based on the title and abstract. All selected articles after this phase were full-text screened and evaluated for eligibility based on the inclusion criteria. Duplicates were identified using Endnote Version 20.

### 2.5. Data items

The following details from the included studies were extracted. These included the ‘authors details’, ‘study design, location, and period,’ ‘population and sampling procedure,’ ‘exposure assessment,’ ‘outcome assessment,’ ‘effect modifier(s) assessed,’ ‘covariates,’ ‘statistical method,’ ‘notes on study design and main effect results’ and ‘summary of major findings regarding effect modification.’

### 2.6. Synthesis of results

These extracted details were summarized in the results (table 1). A qualitative synthesis of the included studies was done. In the qualitative synthesis, the study details were organized based on study design and population characteristics, exposures assessed, outcomes assessed, and effect modifiers assessed. The main findings from the studies were summarized by whether the study explored adverse birth or early childhood health outcomes. Findings concerning effect modification were summarized based on whether the effect modifier examined was maternal BMI or nutrition-related factors and physical activity pattern.

## 3. Results

### 3.1. Selection of source evidence

**Figure 2.**
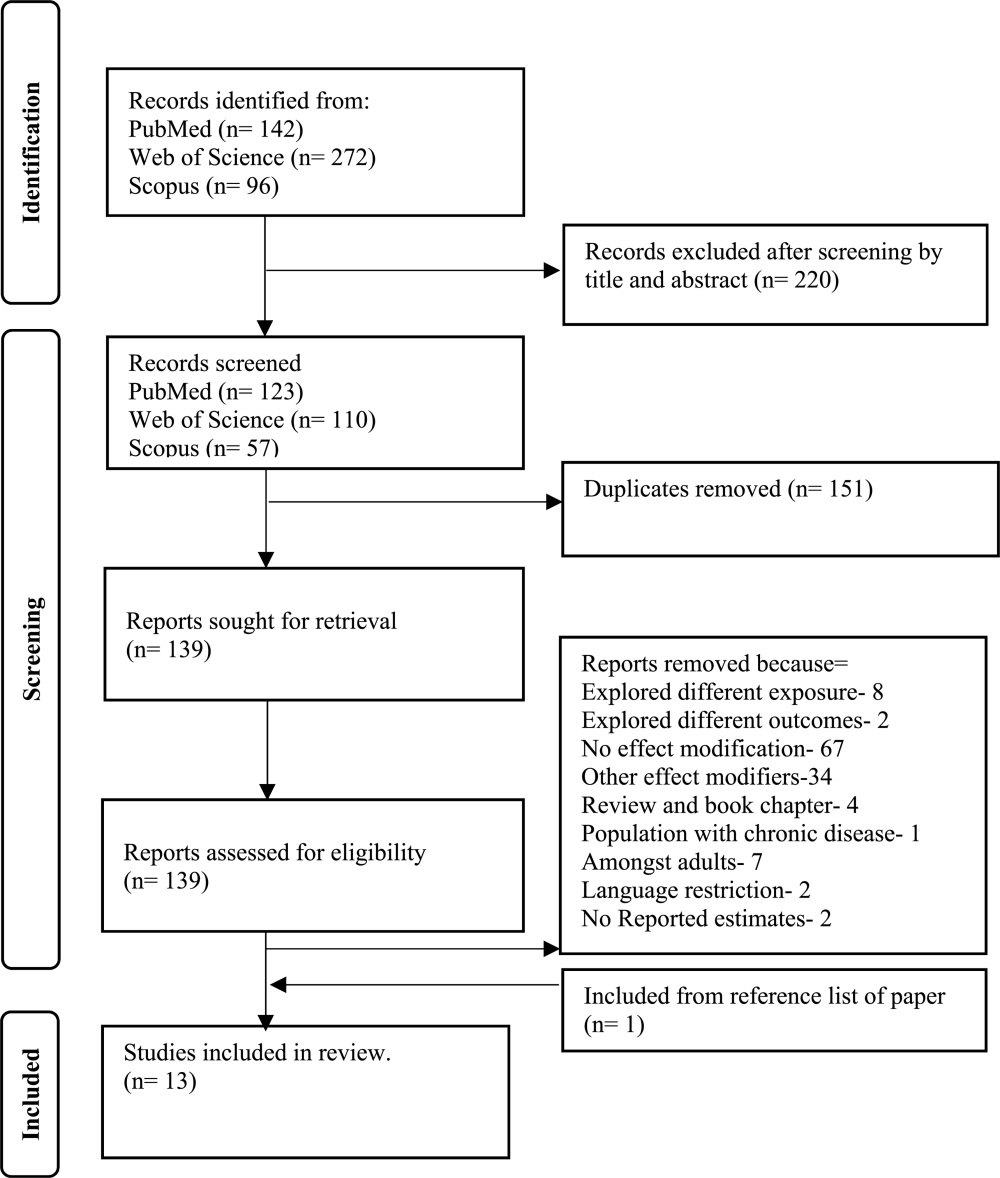
A PRISMA flowchart demonstrating how studies were included in the review.

The initial search carried out in the databases yielded 142, 272, and 96 articles for PubMed, Web of Science, and Scopus, respectively. After screening by title and abstract, a total of 220 articles from all three databases combined were removed. The remaining 290 studies were downloaded, after which 151 duplicates were removed. The remaining 139 studies were further assessed for eligibility, after which 127 were excluded, with 12 included in the scoping review. No new studies were identified via the references of systematic reviews; however, one study was identified from the reference list of one of the included studies. Figure 2 is the PRISMA [27] flowchart indicating the study selection process.

### 3.2. Synthesis of results

#### 3.2.1. Study design and population characteristics.

Study details and characteristics were summarized and presented in table 1. Of the thirteen studies included ten were cohort studies [28, 29, 31, 32, 34–38, 40] (six prospective studies and four retrospective studies), and three were cross-sectional studies [30, 33, 39]. Five of these studies were conducted in China [31–33, 39, 40], four in the USA [35–38], and one each in Israel [28], Mexico [29], and Spain [30]. One study was based on data from the USA and Poland [34]. The majority (10) of these studies were conducted amongst mother-child pairs or singleton births [28, 29, 31, 32, 34–38, 40], whereas 3 were conducted amongst children (as the unit of analysis) [30, 33, 39].

#### 3.2.2. Exposure assessment

In relation to the exposure assessed, twelve of the thirteen studies explored the impact of various air pollutants (PM, NO_2_, ozone [O_3_], ultrafine particles, black carbon [BC], elemental carbon [EC]) [28–39] whereas one study explored the impact of cooking oil emissions on various health outcomes [40]. The majority of studies that assessed exposure via specific pollutants relied on area-specific estimates (residential, school, or city level) [28–33, 35–39] while the study which examined the impact of cooking oil exposure obtained exposure information from a questionnaire [40]. The study by Jedrychowski *et al* [34] assessed air pollution exposure via personal monitoring.

#### 3.2.3. Outcome assessment

Of the thirteen studies, nine focused on adverse birth outcomes such as birthweight, LBW, large for gestational age (LGA), small for gestational age (SGA), PTB, and term low birthweight (which included term births that were born LBW) (TLBW) [28, 29, 31, 32, 35–38, 40] whilst three focused on early child BMI [30, 33, 39]. Details on the majority of the adverse birth outcomes were obtained via medical records whereas BMI measurements were computed based on weight and height measurements.

#### 3.2.4. Effect modifiers assessment

Of the studies included, the most common effect modifier explored was pre-pregnancy BMI, which was examined in 5 of the studies [28, 31, 36, 37, 40]. In all but one of the studies, where maternal BMI was self-reported [34], data on maternal BMI was generally obtained via either health records or through health examination. Two studies explored effect modification by child physical activity [30, 33], whereas one each of the studies explored effect modification by maternal energy-adjusted dietary inflammation score (E-DII) [29], maternal alcohol intake [32], maternal caloric/energy, fat, and saturated fat intakes [35], maternal fish consumption [34], prenatal nutritional supplementation [38], child fruit and vegetable intakes [33], and child dietary problems [39]. In the majority of these studies, data on dietary and physical activity patterns were primarily assessed via an unvalidated questionnaire. Only three studies [29, 34, 35] used a food frequency questionnaire to assess dietary patterns.

### 3.3. Evidence of effect modification

In relation to evidence of effect modification, a large majority of the studies [28–30, 32–34, 36–40] conducted stratified analysis or presented coefficients from models that included a product term and one study explored it by reporting the interaction parameter estimates and interaction contrast ratios to determine whether interaction occurred on the additive scale [35].

#### 3.3.1. Pre-pregnancy BMI

##### 3.3.1.1. Cohort studies

A prospective study conducted in Israel by Ahmad *et al* [28] where PM_2.5_ (per 10-μg/m^3^ increase) exposure was associated with higher odds of TLBW and SGA, the stratum-specific results, contrary to what was hypothesized, showed a decreasing trend with the largest effect estimate reported in the underweight group and lowest in the obese group for both outcomes (TLBW: Underweight: OR = 1.86, 95 CI%: 1.53, 2.26; Normal weight: OR = 1.46, 95% CI: 1.20, 1.78; Overweight: OR = 1.27, 95% CI: 1.04, 1.55; Obese: OR = 1.15, 95 CI%: 0.95, 1.40) (SGA: Underweight: OR = 1.60, 95 CI%: 1.45, 1.76; Normal weight: OR = 1.30, 95% CI: 1.18, 1.43; Overweight: OR = 1.12, 95% CI: 1.01, 1.23; Obese: OR = 1.06, 95 CI%: 0.96, 1.17). Similarly, in the prospective cohort study by Wang *et al* [40], the observed stratum-specific odds ratios amongst maternal pre-pregnancy BMI categories with respect to the impact of 0–1 h day^−1^ (assumed to be > 0–1 h day^−1^) exposure to cooking oil fumes compared to 0 h day^−1^ on LGA were inconsistent, with an increased odds observed only in mothers with normal BMI (OR = 2.30, 95% CI: 1.56,3.40), whilst no evidence of an association was observed for all other categories (Underweight: OR = 0.46, 95% CI: 0.16, 1.38); Overweight: OR = 0.86, 95% CI: 0.27, 2.31; Obese: OR = 0.33, 95% CI: 0.03, 3.20).

In a retrospective cohort study by Laurent *et al* [37], pre-pregnancy BMI appeared to modify the associations between various air pollutants (PM_2.5_ and ozone) and LBW (although authors did not report p-values for interaction); however, the patterns of effect modification were not consistent. For instance, higher exposure to PM_2.5_ (per IQR increase) was associated with a decreased odds of LBW amongst children born to mothers with BMI ⩽ 19.9 kg m^−2^ (OR = 0.868, 95% CI: 0.765, 0.985) and an increased odds amongst children born to mothers with pre-pregnancy BMI > 35 kg m^−2^ (OR = 1.235, 95% CI: 1.037, 1.471). For all the other BMI categories, no evidence of an association was observed (20–24.9 kg m^−2^: OR = 1.041, 95% CI: 0.969, 1.119; 25–29.9 kg m^−2^: OR = 1.016, 95% CI: 0.928, 1.111; 30–34.9 kg m^−2^: OR = 1.139, 95% CI: 0.992, 1.309). Additionally, for ozone, an increase in the pollutant was associated with a decreased odds of LBW amongst children born to mothers with pre-pregnancy BMI ranging from 30 kg m^−2^ to 34.9 kg m^−2^ (OR = 0.912, 95% CI: 0.857, 0.972) while all other BMI categories showed no association (⩽19.9 kg m^−2^: OR = 1.024, 95% CI: 0.964, 1.085; 20–24.9 kg m^−2^: OR = 0.991, 95% CI: 0.959, 1.023; 25–29.9 kg m^−2^: OR = 1.003, 95% CI: 0.964, 1.045; > 35 kg m^−2^: OR = 1.016, 95% CI: 0.937, 1.100). For NO_2_ exposure, no evidence of associations was observed across all categories of maternal pre-pregnancy BMI (⩽19.9 kg m^−2^: OR = 0.976, 95% CI: 0.901, 1.057; 20–24.9 kg m^−2^: OR = 0.985, 95% CI: 0.943, 1.031; 25–29.9 kg m^−2^: OR = 0.986, 95% CI: 0.931, 1.044; 30 k g m^− 2^ –34. 9 kg m^−2^ OR = 1.046, 95% CI: 0.958, 1.141; > 35 kg m^−2^: OR = 1.069, 95% CI: 0.955, 1.196).

In the study by Du *et al* [31], trimester-specific and throughout the entire pregnancy analyses, contrary to the current state of the science, found that higher PM_2.5_ exposure was associated with elevated birthweight with a trend of increasing associations from the mother’s lowest BMI category to the highest. Lakshmanan *et al* [36] conducted a three-way interaction (BC p-interaction = 0.002; PM_2.5_ p-interaction = 0.02) between child sex, maternal BMI, and air pollutants (BC and PM_2.5_); however, the patterns across combinations of the effect modifiers were inconsistent and hypothesized associations were observed in three of the groupings. The authors observed a decrease in birthweight for gestational age (BWGA) z-scores amongst male children born to obese mothers for every IQR increase in BC and PM_2.5_. Among children born to non-obese mothers, an increase in BC was associated with a decrease in BWGA z-scores amongst female children.

All studies exploring the effect-modifying role of maternal pre-pregnancy BMI in the relationship between air pollution and adverse birth outcomes were cohort studies. The effect modification-related findings from these studies were largely suggestive, albeit with inconsistencies in patterns observed across BMI categories.

#### 3.3.2. Nutrition-related factors and physical activity pattern

##### 3.3.2.1. Cohort studies

Several studies explored the impact of various nutritional factors on the associations of interest. In a longitudinal study by Buxton *et al* [29], although the stratum-specific odds were different across energy-adjusted dietary inflammatory index (E-DII) categories, the authors concluded on the absence of evidence supporting the effect modifying role of E-DII on the relationship between PM_10_ exposure on PTB because all the interval estimates contained the null value.

Maternal alcohol consumption as reported in the study by Guo *et al* [32] may have modified the impact of PM_2.5_ exposure on PTB. For exposure levels across the entire duration of pregnancy, the impact of PM_2.5_ exposure (per 10 μg m^−3^) on PTB was highest among the mothers who drank alcohol prior to being pregnant (OR = 1.06, 95% CI: 1.05, 1.07) than those who did not (OR = 1.05, 95% CI: 1.05, 1.06). However, although the p-value for interaction was significant (p-interaction = 0.003), results should be interpreted cautiously as the stratum-specific odds ratios were very similar. This pattern was similar when PM_2.5_ exposure was assessed for only the third trimester although the p-value for interaction was not significant. When PM_2.5_ exposure was defined for the first and second trimesters, the pattern was reversed; the odds of PTB were high amongst non-drinkers but no evidence of an association was observed amongst drinkers (first-trimester OR = 0.98, 95% CI 0.95, 1.01; second-trimester OR = 0.99, 95% CI: 0.96, 1.02); the p-values for interaction were significant in both trimesters.

Based on reported effect estimates, Jardel *et al* [35] found suggestive evidence of effect modification by all gestational parent dietary characteristics (caloric/energy, fat, and saturated fat intakes) in relation to air pollutant exposures (PM_2.5_, ozone, and NO_2_) and PTB. The authors commented that while confidence intervals were wide, the interaction contrast ratios indicate a departure from the additive scale. In the study by Jedrychowski *et al* [34] the differing impact of PM_2.5_ exposure (particularly in the highest tertile) on birthweight across various categories of maternal fish consumption provides suggestive evidence for effect modification.

Rhee *et al* [38] examined the differences in associations between PM_2.5_ exposure (per 1μg/m^3^) during the second trimester and birth weight with respect to mothers’ participation in prenatal nutritional supplementation (participated: −6.9 grams, 95% CI: −18.5, 4.7; did not participate: −18.3 grams, 95% CI: −45.6, 9.1). Increased PM_2.5_ was associated with decreased birthweight, with a larger decrease observed for those not participating in the supplementation program; however, confidence intervals were wide and included the null association for both groups. Although the authors had data on all three trimesters, effect modification results were only presented for the second trimester.

These cohort studies provide suggestive evidence that the impact of air pollution on adverse birth outcomes could potentially be modified by dietary-related factors. Notwithstanding, given the lack of substantial differences observed in some of the stratum-specific effect estimates, such findings must be interpreted cautiously.

##### 3.3.2.2. Cross-sectional studies

Su *et al* [39] reported that the odds of a child being overweight or obese due to outdoor PM_2.5_ (per 10 μg m^−3^ increase) and ozone (per 10 μg m^−3^ increase) levels were high among preschoolers with a dietary problem (PM_2.5_: OR = 1.005, 95% CI: 1.002, 1.009; Ozone: OR = 1.006, 95% CI: 1.001, 1.010). For children with no dietary problem, no associations were observed for the pollutants (PM_2.5_: OR = 1.003, 95% CI: 0.996, 1.010); Ozone: OR = 1.002, 95% CI: 0.994, 1.009). However, all odds ratios were small in magnitude and the stratum-specific odds ratios were quite similar (less than 10% difference) which suggests an absence of meaningful effect modification.

Additionally, in a stratified analysis with respect to the child’s daily fruit intake, daily vegetable intake, and physical activity pattern, Guo *et al* [33] observed relatively higher odds of obesity upon PM_2.5_ exposure (per 10 μg m^−3^) across all tertiles for the respective effect modifiers, except for in the third tertile of physical activity pattern (Vegetable intake:T1: OR = 1.05, 95% CI: 1.03, 1.08, T2: OR = 1.04, 95% CI: 1.02, 1.07, T3: OR = 1.05, 95% CI: 1.03, 1.08; Fruit intake: T1: OR = 1.04, 95% CI: 1.02, 1.07, T2: OR = 1.05, 95% CI: 1.03, 1.08, T3: OR = 1.05, 95% CI: 1.03, 1.08; Physical Activity: T1 = 1.05, 95% CI: 1.03, 1.08, T2 = 1.06, 95% CI: 1.03, 1.08, T3 = 1.03, 95% CI: 0.99, 1.06). However, the effect estimates in all tertiles were very similar, with p-values for interaction not significant (Vegetable intake = 0.68; Fruit intake = 0.13; Physical activity = 0.07), which posits a lack of evidence for effect modification.

In a cross-sectional study by de Bont *et al* [30] although the main analysis highlighted that multiple pollutants (PM_10_, NO_2_, EC, UFP) were associated with increased odds of being overweight or obese, the stratified analysis with respect to child physical activity was inconsistent, with authors concluding on the absence of effect modification.

All the studies focusing on childhood BMI were cross-sectional studies: two studies focused on diet-related effect modifiers, and one focused on physical activity. Both diet-related and physical activity effect modifiers did not provide clear evidence of effect modification.

## 4. Discussion

### 4.1. Summary of evidence

Although there is some suggestion of the modifying role of maternal pre-pregnancy BMI and nutrition-related factors on the association between air pollution exposure and adverse birth outcomes and childhood obesity, the evidence is largely weak and inconsistent. Further, although we hypothesized the same ameliorating impact of modifiers with antioxidant and anti-inflammatory properties on the associations between early-life exposures and all of the outcomes included, there are too few methodological (e.g., data collection) similarities across studies for which to draw precise inference. For example, the most commonly evaluated effect modifiers, pre-pregnancy BMI (n = 5 studies) and child physical activity (n = 2 studies), were evaluated in the context of different health outcomes; the former focusing on associations with various adverse birth outcomes and the latter on childhood obesity. Additionally, all seven studies evaluating the potential for diet-related effect modification utilized different definitions to assign a status or continuum of healthy or unhealthy diets to study participants (i.e., maternal energy-adjusted dietary inflammation score (E-DII) [29], maternal alcohol intake [32], maternal caloric/energy, fat, and saturated fat intakes [35], maternal fish consumption [34], prenatal nutritional supplementation [38], child fruit and vegetable intakes [33], and child dietary problems) [39].

The lack of harmonization across effect modifier definitions and the inconsistencies in evidence for effect modification could be attributed to several other factors. The limited sample sizes in the stratified analyses in some of the studies will reduce the studies’ statistical power to detect effect modification if it is present [29, 34]. As reported by Knol and VanderWeele [41], a large sample size is helpful to detect meaningful interaction, particularly with the magnitudes of effects expected with environmental exposures. In many situations, studies are often only appropriately powered to identify the main effects, with little or no consideration given to the evaluation of effect modification at the study design stage. Hence, effect modification often ends up being relegated to sensitivity analyses. Additionally, measurement errors resulting from the methods used, particularly in exposure and effect modifier assessment, could have impacted the study findings. For example, as is often noted as a limitation of studies evaluating the main effects of air pollution, the majority of the included studies in the scoping review relied on area-specific estimates for assessing exposure, which may not thoroughly reflect personal exposure levels. Also, the use of self-reported diet and physical activity information could likely result in the misclassification of the effect modifier. For instance, in some of the studies, the use of a simple questionnaire (typically an unvalidated questionnaire that assessed dietary intake), instead of a more robust dietary measurement such as a food frequency questionnaire (FFQ), semi-quantitative FFQ, or biomarker, raises concerns of information bias due to misclassification. As noted by Amstrong [42], measurement error in effect modifiers may lead to a diminishing of the observed effect or the creation of spurious association. Furthermore, many studies reported only on the multiplicative scale of interaction; thus, little is known about the potential for additive interaction (only one study reported on this), which is essential for public health intervention [41]. Also, given that these studies were primarily examined within different populations and geographical contexts, it could be possible that the inherent underlying population differences in levels or distributions of the potential effect modifiers could impact the ability to detect a consistent pattern of interaction across studies even if these patterns exist more broadly across populations (e.g., the range of consumption of various dietary components may not overlap or only overlap minimally across geographic and socio-economic contexts).

Regarding the state of the evidence on effect modification, there is very limited summarized evidence on the potential effect-modifying role of various contextual factors in the relationship between air pollution and health outcomes in general. Overall, the majority of existing reviews focused on a broad definition of socioeconomic status (SES). For instance, a systematic review by Heo *et al* [43] investigating the effect-modifying role of maternal SES on the relationship between particulate matter and birth outcomes (risk of PTB and LBW) noted suggestive evidence of a higher risk from particulate matter exposure in infants of African American/black mothers than in infants of other women. The study found weak evidence that PM was higher for infants of mothers with lower educational attainment. Similarly, reviews exploring effect modification [44–48] in the relationship between air pollution exposure and child health outcomes also observed increased risks among those in lower SES (e.g., by income, education attainment, etc) categories compared to higher SES categories. In general, SES is very complex, and multiple lifestyle and behavioral factors, such as diet and physical activity, are likely affected by SES [49–51]. Although some studies have attempted to better understand which components of SES may be responsible for the modifying role (e.g. Heo *et al* [43], with race, income, educational status, and occupation), very few have considered whether specific lifestyle and dietary factors may be contributing to the observed effect modification by SES. One exception is the review by Westergaard *et al* [47], which reported that the association between ambient air pollution and TLBW was higher among women with extreme BMI (>35 kg m^−2^) compared to non-obese women (⩽19 kg m^−2^). Some tangential evidence supports the specific effect modifying role of physical activity and diet on the associations between aisr pollution and adverse health. A prospective study by Li *et al* [52] found that maternal preconception folic acid supplementation ameliorated the risk of particulate matter exposure to PTB. Further, prospective studies conducted among adults [25, 53] found that various dietary patterns (Mediterranean and alternative Mediterranean diet) modified the effect of air pollution exposure on hypertension [53] and cardiovascular disease-related mortality risk [25]. Also, a review by DeFlorio-Barker *et al* [54] reported that for 9 out of 25 studies reviewed, there was evidence of statistical interaction between air pollution exposure and physical activity with respect to various cardiovascular, respiratory, and neurological health outcomes.

### 4.2. Biological plausibility

Prenatal air pollution exposure has been reported to contribute to adverse outcomes through biological mechanisms such as oxidative stress, inflammation, and epigenetic alterations, which pose a significant threat to the developing fetus (figure 1) [55]. For instance, primary studies conducted in human populations have observed that increasing levels of pollutants, such as PM, O_3_, and NO_2,_ were associated with increased oxidative stress and inflammatory markers, which can affect placental development and intrauterine growth [56–58]. Increased levels of oxidative stress in pregnancy due to air pollution exposure contribute to placental malperfusion [55], which leads to an increased risk of adverse pregnancy outcomes such as preeclampsia and SGA [59]. Similarly, an increase in levels of pro-inflammatory markers such as Tumor Necrosis Factor-alpha, Interleukins-1β, 6, and 10 has been reported to lead to PTB [55, 60]. Maternal pre-pregnancy BMI has also been reported to be associated with higher levels of inflammatory markers (IL-6, C-reactive protein), of which some (IL6) have been implicated in adverse birth outcomes, as well as oxidative stress [61–64]. Postnatally, early-life air pollution exposure has also been reported to impact insulin sensitivity, adiposity, and high BMI in children [65, 66].

Postnatal air pollution exposure also exerts adverse effects on early child health outcomes via oxidative stress and inflammatory processes [67]. Healthy nutrition, physical activity practices, and healthy levels of BMI, particularly before and during pregnancy as well as during early life, may alleviate the negative impact of air pollution on fetal and early child health, as these characteristics have been reported to reduce levels of oxidative stress and inflammation. For instance, the consumption of foods rich in fruit and vegetables, which are antioxidant vitamins during pregnancy, has been observed to protect against oxidative stress by increasing levels of antioxidants [68, 69]. Similar healthy dietary patterns such as adhering to Dietary Approaches to Stop Hypertension (DASH) in pregnancy have been reported to be negatively correlated with inflammation [70]. Amongst children, physical activity has also been reported to be negatively associated with oxidative stress and inflammatory marker levels [71–73]. In fact, various primary studies conducted on the human population have found these lifestyle characteristics, particularly diet and antioxidant supplementation, to reduce the effect of air pollution on various health outcomes [22, 24, 25].

### 4.3. Strengths and limitations

A strength of this scoping review is that it provides the state of the evidence surrounding the potential effect modifying role of pre-pregnancy BMI, nutrition, and physical activity on air pollution and adverse birth and childhood outcomes, a topic that is difficult to summarize. Evidence regarding this research question is particularly challenging to capture as the presentation of effect modification and interaction is often not consistent (e.g., different terminologies are used and authors focus on the results in various ways, from highlighting the evidence in the manuscript title or abstract to including results stratified by potential modifiers only in supplemental materials). Thus, although we used a liberal approach to capture the evidence, it is possible that some studies were missed. This review is also affected by several other limitations. The outcomes included in the review occur at different developmental stages (at birth or early childhood) and, therefore, may differ in regard to downstream mechanisms of outcome development. However, we chose to combine these outcomes because of the similar timing of the exposures and because similar biological pathways likely underlie the interacting effect of air pollution and our selected effect modifiers (i.e., oxidative stress and systemic inflammation) early in the development of these adverse health outcomes. Also, there are limited numbers of studies on the individual outcomes, thus, by combining these outcomes, we were able to describe the state-of-the-science and characterize effect modification by these factors that are relevant to both sets of outcomes. Here we focused on nutrition and physical activity-related effect modifiers because of their role in affecting oxidative stress and inflammation; there are other potential effect modifiers such as SES-related factors, sleep changes, and psychological changes, which we did not cover and may also affect air pollutant-health associations via stress-related mechanisms. By including only studies published in the English language, we may miss equally important contributions to the literature published in other languages. Further, in terms of methodological bias, since this study is a scoping review, no attempts were made to assess the methodological quality of the included studies. As more evidence is published, ideally with cohesion among definitions, it will be important to conduct a systematic review of the literature.

## 5. Conclusion and recommendations

Our review concluded that evidence is inconsistent on the potential effect modifying role of nutrition, physical activity patterns, and pre-pregnancy BMI on the relationship between air pollution and adverse birth and early-life health outcomes. There is an urgent need for rigorous air pollution-related studies to explore effect modification since a better understanding of the heterogeneity of main effect associations may lead to targeted policy solutions for addressing the health impact of air pollution. In designing these studies, we recommend appropriate sample sizes to ensure that studies are sufficiently powered to detect effect modification, if there is any, and adopting robust measurements of potential modifiers to minimize the potential for misclassification. For instance, dietary effect modifiers should be measured with validated FFQs, whereas physical activity should be assessed via precise technical devices and standardized questionnaires. Also, for the body of evidence on effect modification to be established, the reporting of effect modification results should be more consistent across studies, including reporting on the scale (additive or multiplicative) on which the effect modification is occurring (e.g., see Knol and VanderWeele [41]). Understanding the nature of these interactions, particularly on the additive scale, must be prioritized to provide insight into the public health significance of these modifiable characteristics [41]. Finally, there is also the need for more rigorous prospective studies, particularly in low-resource areas, to help improve contextual understanding of the potential for effect modification, especially by behaviorally modifiable risk factors, in air pollution research.

## Figures and Tables

**Table 1.** Characteristics of included studies.

Author Details	Study design, location, and period	Population and sampling procedure	Exposure assessment	Outcome assessment	Effect modifier(s) assessed^c^	Covariates	Statistical Method	Notes on study design and main effect results	Summary of Major Findings Regarding Effect Modification

Ahmad *et al* [28]	Population-based retrospective cohort. Israel 2004 to 2015	381265 Singleton live births.	Daily geocoded maternal residential PM_2.5_ levels were used to estimate levels during the entire duration of pregnancy as well as trimester-specific levels using gestational length.	Term low birth weight (TLBW) (weight ⩽2500g and gestational age ⩾37weeks) and small for gestational age (SGA) (<10th percentile of birth weight for gestational age). Obtained from maternal health records.	Pre-pregnancy body mass index (BMI). Obtained from maternal health records	Infant sex, year and season of birth, gestational age, birth order, maternal age at child’s birth, maternal height, maternal smoking before conception, Maternal locality of residence and administrative census area, and socio-economic status (SES) score. Assessed via multiple records.	Multilevel logistic regression with random effects for maternal locality of residence, administrative census area, and mother.	PM_2.5_ exposure during the entire pregnancy was associated with increased odds of TLBW and SGA. Did not report p-interaction. The study employed no personal monitoring. Outcome and effect modifier information was obtained from reliable sources (medical records).	The differing stratum-specific odds ratios provided suggestive evidence of maternal pre-pregnancy BMI modifying the relationship between PM_2.5_ exposure during pregnancy and TLBW and SGA. The stratum-specific risk estimates decreased monotonically between the underweight and obese categories.
Buxton *et al* [29]	Longitudinal (Cohort) Study. Mexico, 1994–2005	1216 pregnant women recruited at either delivery or during pregnancy.	PM_10_ estimates were generated from daily citywide averages. Obtained trimester-specific averages and averages over entire pregnancy	Preterm birth (PTB)-birth less than 37 weeks); Gestational age was assessed based on last menstrual period.	Maternal dietary intake was assessed using semi-quantitative FFQ. Estimated energy-adjusted dietary inflammatory index (E-DII)	Mother’s age at enrollment, parity, years of education, cohort, day of year (using natural splines), temperature (using natural splines) and precipitation (using natural splines), and marital status. Assessed via questionnaire	Cox proportional hazards model.	PM_10_ exposure was associated with an increased risk of PTB during the second trimester. Did not report p-interaction value. The study employed no personal monitoring. Effect modifier information was obtained via questionnaire, while outcome data was obtained via maternal self-reporting and data from the clinical team.	There was no clear evidence supporting the effect modifying role of E-DII on the relationship between PM_10_ exposure and childhood obesity.
de Bont *et al* [30]	Cross-sectional study. Barcelona-Spain, 2012	2660 children aged 7–10 years during 2012 from forty primary schools. 59% participation rate	Land use regression (LUR) models were used to estimate levels of nitrogen dioxide (NO_2_), particulate matter levels at home. Outdoor levels of NO2, PM_2.5_, elemental carbon (EC), and ultrafine particles (UFP) were measured in the schoolyard.	Child weight and height were measured, and age- and sex-specific z-scores for body mass index (zBMI) were calculated using the WHO growth reference 2007. Overweight and obesity were defined using the same reference.	Child physical activity. Assessed via parents’ questionnaire	Maternal and paternal education, maternal and paternal country of birth, paternal employment status, number of siblings, household status and maternal smoking during pregnancy, and SES. Assessed via parents’ questionnaire	Multilevel mixed linear and ordered logistic model.	For school-level NO2, PM_2.5,_ and EC, compared to children in the lowest tertile of exposure, children in the second tertile of exposure were at higher odds of being overweight or obese than having a normal weight. The study employed no personal monitoring. Covariate and effect modifier information were obtained via questionnaire.	There was no evidence supporting the effect modifying role of child physical activity on the relationship between home (PM_10_) and school (NO_2_, PM_2.5_ and EC) pollutant exposures and childhood obesity.
Du *et al* [31]	Prospective Cohort Study China, January 1, 2010 to December 31, 2012.	193461 Chinese women from 220 counties	Gestational PM_2.5_ was derived from a hindcast model for historical PM_2.5_ estimation from satellite-retrieved aerosol optic depth. County-level PM_2.5_ was extrapolated to obtain trimester averages.	Birth weight (grams (g)) as continuous measure. Obtained from health records	Pre-pregnancy BMI measured preconception. Categorized into underweight, normal, overweight and obese. Assessed via health examination.	Neonate’s sex, maternal age, gestational week, educational level, maternal smoking or alcohol intake during pregnancy, multiparity, pre-pregnancy diabetes mellitus, pre-pregnancy hypertension, birth weight, the season of delivery. Assessed via questionnaire prior to pregnancy	Multivariable linear regression.	Across the entire pregnancy PM_2.5_ was associated with increase in birth weight. Trimester-specific estimates were consistent. The study employed no personal monitoring. Covariate information was obtained prior to pregnancy via a questionnaire.	Maternal pre-pregnancy BMI modified the relationship between PM_2.5_ exposure during the first and second trimesters and the entire duration of pregnancy and birth weight. The trend of the relationship between PM_2.5_ exposure and birthweight increased across increasing BMI categories.
Guo *et al* [32]	Retrospective Cohort Study, China, January 2014 and December 2014	426246 single birth pregnancies from 129 cities from 30 provinces in China.	Residential PM_2.5_ exposure was obtained from daily city average. Extrapolated to obtain trimester specific averages.	Preterm birth was defined as delivery before 37 weeks of gestation. Gestational age was ascertained based on the last menstrual period and date of delivery. Assessed via health records.	Maternal alcohol use assessed via questionnaire prior to pregnancy.	Maternal age, education level, occupation, second-hand smoking, alcohol use, pre-pregnancy BMI, baby’s sex, number of previous pregnancies, coastal areas and season of conception. Assessed via questionnaire prior to pregnancy	Cox proportional hazards regression	Across the entire pregnancy PM_2.5_ was associated with an increased odd of PTB. Trimester-specific estimate was consistent. The study employed no personal monitoring. Effect modifier and covariate information were obtained prior to pregnancy via a questionnaire.	Despite significant p-interaction, the evidence supporting the effect modifying role of maternal alcohol intake on the relationship between PM_2.5_ exposure during the first, second trimesters and the entire duration of pregnancy and PTB was not conclusive because the stratum-specific odds ratios were similar.
Guo *et al* [33]	Cross-sectional, China 2013–2014	40953 school-children aged 6–17 years. Multi-stage stratified sampling 97.2% participation rate.	5-year average PM_2.5_ exposure was estimated as the concentration at the school location for each participant.	BMI (kg/m2) was measured physician beam scale with a height rod. Categorized as normal, overweight, and obese for their sex and age group, according to the Chinese national standard Screening for Overweight and Obesity among school-age children and adolescents	Child fruit and vegetable intake and physical activity was assessed via questionnaire^c^	Gender, Age, Residence, school type, maternal education level, maternal occupation, economic level, ventilation, household cooking fuel, School heating fuel type, Activity time, second smoke. All were assessed via the questionnaire	Weighted logistic regression models.	Increasing PM_2.5_ exposure was associated with increased odds of being obese. Long-term exposure assessment and no personal monitoring. Effect modifier and covariate information was obtained via questionnaire.	There was no evidence to support the effect modifying roles of child physical activity pattern, daily fruit intake, and daily vegetable intake on the relationship between PM_2.5_ exposure at school and BMI.
Jedrychowski *et al* [34]	Prospective cohort study. New York City (USA) and Krakow (Poland). January 2001 and February 2004.	481 nonsmoking women with singleton pregnancies, of 18–35 years of age, who gave birth at term.	Personal PM_2.5_ monitoring in second trimester (48 h average. Stratified by tertile.	Birth weight. Obtained from maternal health record. The gestational age at birth was defined as the interval between the first day of the mother’s last menstrual period and the date of birth.	Maternal dietary frequency (never, less than once a month, once a week, 1–2 times a week, 3–4 times a week or every day) of consumption of smoked, fried, roasted and grilled fish servings in second and third trimester. Assumed that each fish meal averaged 150 g to estimate the median grams of fish eaten per week. Assessed via semiquantitative FFQ	Maternal age, maternal education, maternal height, maternal pre-pregnancy BMI, gestational age, parity, child sex, fish consumption. Assessed via maternal health records and via questionnaire	Linear regression	Compared to those with low exposure, mothers with higher exposure were at higher risk of having children with lower birthweight. Personal monitoring reduces the risk of measurement error	There was evidence to support the effect modifying role of maternal fish consumption on the relationship between PM_2.5_ and birthweight. The association between PM exposure and child birthweight increased across the categories of fish intake, from low to high consumption.
Jardel *et al* [35]	Prospective Cohort study, United States of America, 2009 to 2011	1505 Gestational parent and offspring pairs	Average daily pollutants [O_3_, PM_2.5_ (two set of values from two models; EPA’s Fused Community Multiscale Air Quality model and an ensemble model), and NO_2_ exposure] geocoded to maternal address at enrolment and estimated for each trimester. Pollutants are represented as follows: O_3_ exposure was represented as 8-hour maxima in ppb, PM_2.5_ as 24-h averages in μg/ m^3^, and NO_2_ as1 h maxima in ppb.	PTB defined as birth before 37 weeks of gestation. Information was abstracted from medical records either based on clinical estimate or LMP or date of delivery.	Gestational parent dietary pattern (6 months prior to pregnancy) assessed via FFQ. Energy/caloric intake (kcal), fat intake (%) and saturated fat intake (%)	Gestational parent age at delivery, gestational parent race, education, pre-pregnancy BMI, household income, conception season. Obtained via questionnaire	Log binomial regression	No associations were observed across trimester-specific pollutant exposures pollutants [O_3_,PM_2.5_, and NO_2_ exposure] and PTB. The study employed no personal monitoring. Diet and covariate information was obtained via questionnaire. Reported no p-interaction values	There was no conclusive evidence to support effect modification. The authors reported slight departure from additive scale due to inconsistencies in interaction contrast ratios estimates, albeit all associations were null.
Lakshmanan *et al* [36]	Prospective Cohort Study, USA August 2002-September 2009	670 urban ethnically mixed mother–child pairs.	Black carbon (BC) levels were estimated using validated a spatiotemporal land-use regression (LUR) model; fine particulate matter (PM_2.5_) was estimated using a hybrid LUR model incorporating Satellite-derived Aerosol Optical Depth measures. Individual daily BC and PM_2.5_ exposure level was assigned to participant’s address during pregnancy and used to estimate levels throughout the entire pregnancy.	Birth weight (g) was obtained from labor and delivery record. Estimated birth weight for gestational age zscore (BWGA)	Maternal pre-pregnancy height and weight were ascertained by self-report at enrollment.	Season of birth, maternal race, education, age at enrollment, prenatal smoking, prenatal NLEs, and neighborhood disadvantage index. Ascertained via questionnaire	Stratified multivariable linear regression model.	Both increasing levels BC and PM_2.5_ exposure were associated with decrease in BWGA-zscores. No personal monitoring. Effect modifier and covariate information was obtained via questionnaire.	There was some evidence supporting the potential effect modifying role of maternal BMI and child sex on the relationship between PM_2.5_ and BC exposures and estimated birth weight for BWGA-zscores. The hypothesized association (increase pollution associated with decreased BWGA-zscores) was observed only among boys born to obese mothers (for both PM_2.5_ and BC exposure) and among girls born to non-obese mothers (for BC exposure).
Laurent *et al* [37]	Retrospective Cohort study Los Angeles County, USA January 1, 2001 to December 31,2008	Birth certificate records for all births	Geocoded- residential particulate matter (PM) concentrations. Measured fine PM, nitrogen dioxide and ozone concentrations were interpolated using empirical Bayesian kriging. Measurements were extrapolated for entire pregnancy and all trimesters	Low birth weight data obtained from birth records.	Maternal BMI obtained from health records.	Maternal race/ethnicity, education, parity, trimester primary care beginning, infant’s gender, maternal age, length of gestation and median income by census Block Group. Assessed from health records	Generalized additive models logistic link function with a quasi- binomial distribution	Increasing air pollutants exposure was associated with increased odds of being LBW. Did not report p-interaction. Long-term exposure assessment and no personal monitoring. Covariate information was obtained from health records.	There was no evidence to support the effect modifying role of maternal BMI categories on the risk of LBW upon pollutants exposure.
Rhee *et al* [38] ^a^	Retrospective cohort study, USA 2007–2015	3366 birth records	Daily PM2.5 concentration to each residential address. Estimated trimester specific averages using date of birth and gestational age (in weeks).	Birthweight (g) abstracted from electronic health and survey (was used if birthweight was missing heath records) records.	Maternal participation in nutritional supplementation program from questionnaire-based survey records.	Maternal age, BMI, race/ethnicity, nativity, education, smoking, insurance, marital status, child gestational age and sex, seasonality, and block-group-level median household income. All obtained from multiple health records	Linear regression models.	Increasing PM_2.5_ exposure was associated with reduced birthweight across entire pregnancy and in the second and third trimester. Did not report p-interaction. No personal monitoring. Outcome, effect modifier, and covariate information were obtained from survey.	Despite the differing stratum-specific effect estimate, there is no clear evidence of effect modification by prenatal nutritional supplementation participation.
Su *et al* [39]	Cross-sectional study, Anhui Province (China), September 2019 to January 2020	3636 preschool children (3–6 year) from 26 kindergartens in 4 rural areas 95.63% response rate	Outdoor air pollutants (PM_2.5_ and O_3_) were derived by matching preschoolers’ external air pollution exposure according to their kindergarten address or neighborhoods or administrative villages	Obesity indicators were evaluated according to the World Health Organization BMI criteria for preschool children. Outcome of interest overweight/obese. Assessed via physical examination	Dietary problems (Eating behavior problems). Assessed via questionnaire^c^	Age, gender, father and mother education level, family monthly net income, left-behind status, birth weight, outdoor exercise times, and dietary habits. Assessed via questionnaire	Generalized linear model with logistic function.	Increasing PM2.5 exposure was associated with increased odds of being overweight/obese. Did not report p-interaction. No personal monitoring. Effect modifier and covariate information was obtained via questionnaire.	Although stratified analysis indicates highest odds amongst children with dietary problems there was no evidence of effect modification to conclusively support the role of dietary problems in the relationship between PM_2.5_ exposure and childhood obesity.
Wang *et al* [40] ^b^	Prospective Cohort study Nanjing (China) 2013	420 pregnant women from 2013 to 2015 and follow up for one year until the offspring. 95.5% participation rate	Information of the cooking oil fume exposure was obtained from the early pregnancy questionnaire. Exposure status was categorized into non-exposed group and exposed group or three exposure time subgroups, including 0 h /day, 0–1 h day^−1^ and > 1 h day^−1^ respectively.	LBW was defined as birth weight 2500 g. Additionally, birth weight was also defined as SGA (below the 10th percentile,), appropriate for gestational age AGA- (as between the 10th and 90th percentile) and large for gestational age (LGA) (birth weight higher than 90th percentiles of gestational age sex-specific birth weight based on the birth weight reference percentiles for Chinese.	Pre-pregnancy BMI was obtained from obstetric records.	Maternal age, pre-pregnancy BMI, maternal education level, maternal smoking status, income, parity and gestational diabetes. Assessed via face-to-face qsuestionnaires in the first trimester (12weeks of pregnancy), 24-week antenatal (during second trimester) and 32-week antenatal (during third trimester) and via obstetric records.	Multinomial logistic regression model	**Compared to non-exposed group, mothers reporting 0–1 hrday**^−**1**^ **of exposure were at an increased odds of having LGA children LGA, whereas no association was observed among mothers in the highest exposure** (>**1 h day^−1^**). **Did not report p-interaction. No personal monitoring. Outcome and covariate information was obtained via questionnaire.**	There was no evidence of effect modification by pre-pregnancy BMI in the relationship between cooking oil fume exposure and LGA. Stratified analysis indicates an increase odds of LGA amongst mothers with normal BMI, whilst the relationship in the extreme groups (underweight, overweight, and obese) showed no association.

FFQ- food frequency questionnaire; PTB- Preterm birth; BW- Birthweight; BWGA- Birth weight for gestational age; LBW- Low birth weight; TLBW- Term low birthweight; SGA- Small for gestational age; BMI- Body mass index; LGA-Large for gestational age; EC- Elemental carbon; UFP- Ultrafine particles; BC- Black carbon; SES- Socio-economic status.

a The authors only conducted effect modification for the second trimester.

b Though the authors reported multiple effect estimates for the various outcomes, stratified analysis was only reported for LGA (therefore this outcome was the focus).

c Unless stated otherwise, dietary assessment undertaken via simple questionnaire (typically an unvalidated questionnaire that assesses dietary intake).

## Data Availability

No new data were created or analysed in this study.

## Abbreviations

BC: Black carbon
BMI: Body mass index
CI: Confidence interval
EC: Elemental carbon
E-DII: Energy-adjusted dietary inflammation index
FFQ: Food frequency questionnaire
LGA: Large-for-gestational age
LBW: Low birthweight
OR: Odds ratio
PM: Particulate matter
PTB: Preterm birth
SES: Socio-economic status
SGA: Small-for-gestational age
TLBW: Term low birthweight
UFP: Ultra fine particles
UI: Uncertainty level

## References

[1] WHO. Ambient (outdoor) air pollution. Published 2022 Accessed 2023 https://who.int/news-room/fact-sheets/detail/ambient-(outdoor)-air-quality-and-health

[2] Health Effects Institute, Global Burden of Disease Study 2019 IHME (2020). State of Global Air 2020 (Published 2020) Accessed 2020 https://stateofglobalair.org/data/#/air/plot

[3] Ghosh R, Causey K, Burkart K, Wozniak S, Cohen A and Brauer M 2021 Ambient and household PM2.5 pollution and adverse perinatal outcomes: A meta-regression and analysis of attributable global burden for 204 countries and territories PLoS Med. 18 e1003718 34582444 10.1371/journal.pmed.1003718 PMC8478226

[4] Ju L 2021 Maternal air pollution exposure increases the risk of preterm birth: Evidence from the meta-analysis of cohort studies Environ. Res 202 111654 34252430 10.1016/j.envres.2021.111654

[5] Nyadanu SD 2022 Prenatal exposure to ambient air pollution and adverse birth outcomes: an umbrella review of 36 systematic reviews and meta-analyses Environ. Pollut 306 119465 35569625 10.1016/j.envpol.2022.119465

[6] Luo M 2023 Household polluting cooking fuels and adverse birth outcomes: an updated systematic review and meta-analysis Frontiers in Public Health 11 978556 36935726 10.3389/fpubh.2023.978556 PMC10020710

[7] Amegah AK, Quansah R and Jaakkola JJK 2014 Household air pollution from solid fuel use and risk of adverse pregnancy outcomes: a systematic review and meta-analysis of the empirical evidence PLoS One 9 10.1371/journal.pone.0113920 PMC4252082 25463771

[8] Parasin N, Amnuaylojaroen T and Saokaew S 2021 Effect of air pollution on obesity in children: a systematic review and meta-analysis Children 8 327 33922616 10.3390/children8050327 PMC8146513

[9] Huang C, Li C, Zhao F, Zhu J, Wang S and Sun G 2022 The association between childhood exposure to ambient air pollution and obesity: a systematic review and meta-analysis Int. J. Environ. Res. Public Health 19 8 10.3390/ijerph19084491 PMC9030539 35457358

[10] Fuller CH, Feeser KR, Sarnat JA and O’Neill MS 2017 Air pollution, cardiovascular endpoints and susceptibility by stress and material resources: a systematic review of the evidence Environ Health 16 58 10.1186/s12940-017-0270-0 PMC5471931 28615066

[11] Laurent O, Bard D, Filleul L and Segala C 2007 Effect of socioeconomic status on the relationship between atmospheric pollution and mortality J. Epidemiol Community Health 61 665–75 17630363 10.1136/jech.2006.053611 PMC2652988

[12] Weichenthal S, Hoppin JA and Reeves F 2014 Obesity and the cardiovascular health effects of fine particulate air pollution Obesity 22 1580–9 24639433 10.1002/oby.20748 PMC4238790

[13] Chia AR 2019 Maternal dietary patterns and birth outcomes: a systematic review and meta-analysis Advances in Nutrition 10 685–95 31041446 10.1093/advances/nmy123 PMC6628847

[14] da Silva S G, Ricardo LI, Evenson KR and Hallal PC 2017 Leisure-time physical activity in pregnancy and maternal-child health: a systematic review and meta-analysis of randomized controlled trials and cohort studies Sports Med 47 295–317 27282925 10.1007/s40279-016-0565-2

[15] Godoy-Cumillaf A 2020 The effects of physical activity and diet interventions on body mass index in latin american children and adolescents: a systematic review and meta-analysis Nutrients 12 1378 10.3390/nu12051378 PMC7284900 32408483

[16] Vats H, Saxena R, Sachdeva MP, Walia GK and Gupta V 2021 Impact of maternal pre-pregnancy body mass index on maternal, fetal and neonatal adverse outcomes in the worldwide populations: a systematic review and meta-analysis Obes. Res. Clin. Pract 15 536–45 34782256 10.1016/j.orcp.2021.10.005

[17] Yu Z, Han S, Zhu J, Sun X, Ji C and Guo X 2013 Pre-pregnancy body mass index in relation to infant birth weight and offspring overweight/obesity: a systematic review and meta-analysis PLoS One 8 e61627 23613888 10.1371/journal.pone.0061627 PMC3628788

[18] Mamluk L 2017 Low alcohol consumption and pregnancy and childhood outcomes: time to change guidelines indicating apparently ‘safe’ levels of alcohol during pregnancy? a systematic review and meta-analyses BMJ Open 7 e015410 10.1136/bmjopen-2016-015410 PMC5642770 28775124

[19] Kannan S, Misra DP, Dvonch JT and Krishnakumar A 2006 Exposures to airbone particulate matter and adverse perinatal outcomes: A biologically plausible mechanistic framework for exploring potential effect modification by nutrition Environ. Health Perspect 114 1636–42 17107846 10.1289/ehp.9081 PMC1665414

[20] Jochum F 2022 Burden of early life obesity and its relationship with protein intake in infancy: the middle east expert consensus Pediatr. Gastroenterol. Hepatol. Nutr 25 93–108 35360379 10.5223/pghn.2022.25.2.93 PMC8958054

[21] Lanigan J 2018 Prevention of overweight and obesity in early life Proc. Nutr. Soc 77 247–56 29808786 10.1017/S0029665118000411

[22] Romieu I 2002 Antioxidant supplementation and lung functions among children with asthma exposed to high levels of air pollutants Am. J. Respir. Crit. Care Med 166 703–9 12204869 10.1164/rccm.2112074

[23] Snow SJ 2019 The influence of maternal and perinatal high-fat diet on ozone-induced pulmonary responses in offspring Journal of Toxicology and Environmental Health, Part A 82 86–98 30755101 10.1080/15287394.2018.1564101 PMC10926063

[24] Amegah AK, Sewor C, Obeng AA, Coker ES and Eliason S 2021 Vitamin D intake modifies the association of household air pollution exposure with maternal disorders of pregnancy Indoor Air 32 1–13 10.1111/ina.12963 34837417

[25] Lim CC 2019 Mediterranean diet and the association between air pollution and cardiovascular disease mortality risk Circulation 139 1766–75 30700142 10.1161/CIRCULATIONAHA.118.035742 PMC6453737

[26] Peters MDJ, Godfrey CM, Khalil H, McInerney P, Parker D and Soares CB 2015 Guidance for conducting systematic scoping reviews JBI Evidence Implementation 13 141–146 10.1097/XEB.0000000000000050 26134548

[27] Tricco AC 2018 PRISMA extension for scoping reviews (PRISMA-ScR): checklist and explanation Ann. Intern. Med 169 467–73 30178033 10.7326/M18-0850

[28] Ahmad WA 2022 Mother-level random effect in the association between PM(2.5) and fetal growth: A population-based pregnancy cohort Environ. Res 210 112974 10.1016/j.envres.2022.112974 35192805

[29] Buxton MA 2020 Particulate matter exposure, dietary inflammatory index and preterm birth in Mexico city, Mexico Environ. Res 189 109852 10.1016/j.envres.2020.109852 PMC7525039 32979989

[30] de Bont J 2019 Ambient air pollution and overweight and obesity in school-aged children in Barcelona, Spain Environ. Int 125 58–64 10.1016/j.envint.2019.01.048 PMC6380992 30703612

[31] Du H 2022 Interaction of PM(2.5) and pre-pregnancy body mass index on birth weight: a nationwide prospective cohort study Front Endocrinol (Lausanne) 13 963827 35957820 10.3389/fendo.2022.963827 PMC9360486

[32] Guo T 2018 The association between ambient PM2.5 exposure and the risk of preterm birth in China: A retrospective cohort study Sci. Total Environ 633 1453–9 10.1016/j.scitotenv.2018.03.328 29758897

[33] Guo Q 2020 Association between exposure to fine particulate matter and obesity in children: a national representative cross-sectional study in China Environ. Int. 143 105950 10.1016/j.envint.2020.105950 32673910

[34] Jedrychowski W 2010 Higher fish consumption in pregnancy may confer protection against the harmful effect of prenatal exposure to fine particulate matter Annals of Nutrition and Metabolism 56 119–26 20134157 10.1159/000275918 PMC2842166

[35] Jardel H, Martin CL, Hoyo C and Rappazzo KM 2023 Interplay of gestational parent exposure to ambient air pollution and diet characteristics on preterm birth BMC Public Health 23 822 37143049 10.1186/s12889-023-15676-x PMC10161541

[36] Lakshmanan A 2015 Associations between prenatal traffic-related air pollution exposure and birth weight: Modification by sex and maternal pre-pregnancy body mass index Environ. Res 137 268–77 10.1016/j.envres.2014.10.035 PMC4354711 25601728

[37] Laurent O 2014 Sources and contents of air pollution affecting term low birth weight in Los Angeles County, California, 2001–2008 Environ. Res 134 488–95 25085846 10.1016/j.envres.2014.05.003

[38] Rhee J 2019 Effects of maternal homelessness, supplemental nutrition programs, and prenatal PM(2.5) on birthweight Int. J. Environ. Res. Public Health 16 10.3390/ijerph16214154 PMC6862522 31661898

[39] Su W 2022 The effect of air pollution and emotional and behavioral problems on preschoolers’ overweight and obesity Environmental Science and Pollution Research 29 75587–96 35657543 10.1007/s11356-022-21144-7

[40] Wang L 2018 The association between cooking oil fume exposure during pregnancy and birth weight: a prospective mother-child cohort study Sci. Total Environ 612 822–30 28881305 10.1016/j.scitotenv.2017.08.031

[41] Knol MJ and VanderWeele TJ 2012 Recommendations for presenting analyses of effect modification and interaction International Journal of Epidemiology 41 514–20 22253321 10.1093/ije/dyr218 PMC3324457

[42] Armstrong BG 1998 Effect of measurement error on epidemiological studies of environmental and occupational exposures Occup. Environ. Med 55 651–6 9930084 10.1136/oem.55.10.651 PMC1757516

[43] Heo S, Fong KC and Bell ML 2019 Risk of particulate matter on birth outcomes in relation to maternal socio-economic factors: a systematic review Environ. Res. Lett 14 123004 10.1088/1748-9326/ab4cd0 PMC8186490 34108997

[44] Burte E, Nadif R and Jacquemin B 2016 Susceptibility factors relevant for the association between long-term air pollution exposure and incident asthma Curr. Environ. Health Rep 3 23–39 26820569 10.1007/s40572-016-0084-1

[45] Munoz-Pizza DM, Villada-Canela M, Reyna MA, Texcalac-Sangrador JL and Osornio-Vargas ÁR 2020 Air pollution and children’s respiratory health: a scoping review of socioeconomic status as an effect modifier Int. J. Public Health 65 649–60 32405779 10.1007/s00038-020-01378-3

[46] Vesterinen HM, Morello-Frosch R, Sen S, Zeise L and Woodruff TJ 2017 Cumulative effects of prenatal-exposure to exogenous chemicals and psychosocial stress on fetal growth: Systematic-review of the human and animal evidence PLoS One 12 e0176331 10.1371/journal.pone.0176331 PMC5507491 28700705

[47] Westergaard N, Gehring U, Slama R and Pedersen M 2017 Ambient air pollution and low birth weight - are some women more vulnerable than others? Environ. Int 104 146–54 10.1016/j.envint.2017.03.026 28390661

[48] Rodriguez-Villamizar LA, Berney C, Villa-Roel C, Ospina MB, Osornio-Vargas A and Rowe BH 2016 The role of socioeconomic position as an effect-modifier of the association between outdoor air pollution and children’s asthma exacerbations: an equity-focused systematic review Rev. Environ. Health 31 297–309 27227707 10.1515/reveh-2016-0005

[49] Ke Y, Shi L, Peng L, Chen S, Hong J and Liu Y 2022 Associations between socioeconomic status and physical activity: a cross-sectional analysis of Chinese children and adolescents Front Psychol. 13 904506 36118481 10.3389/fpsyg.2022.904506 PMC9477139

[50] Kell KP, Judd SE, Pearson KE, Shikany JM and Fernández J 2015 Associations between socio-economic status and dietary patterns in US black and white adults Br. J. Nutr 113 1792–9 25869232 10.1017/S0007114515000938 PMC4597887

[51] Oude Groeniger J, Kamphuis CBM, Mackenbach JP, Beenackers MA and van Lenthe F J 2019 Are socio-economic inequalities in diet and physical activity a matter of social distinction? A cross-sectional study Int. J. Public Health 64 1037–47 31187165 10.1007/s00038-019-01268-3 PMC6677869

[52] Li Q 2020 Folic acid supplementation and the association between maternal airborne particulate matter exposure and preterm delivery: a national birth cohort study in China Environ. Health Perspect 128 127010 33337244 10.1289/EHP6386 PMC7747880

[53] Huang K 2023 Association of fine particulate matter and its constituents with hypertension: the modifying effect of dietary patterns Environmental Health 22 55 10.1186/s12940-023-01000-y PMC10411005 37553681

[54] DeFlorio-Barker S, Lobdell DT, Stone SL, Boehmer T and Rappazzo KM 2020 Acute effects of short-term exposure to air pollution while being physically active, the potential for modification: a review of the literature Preventive Medicine 139 106195 32652130 10.1016/j.ypmed.2020.106195 PMC8043242

[55] Fussell JC, Jauniaux E, Smith RB and Burton GJ 2024 Ambient air pollution and adverse birth outcomes: a review of underlying mechanisms BJOG 131 538–50 38037459 10.1111/1471-0528.17727 PMC7615717

[56] Rossner P 2011 Genetic, biochemical, and environmental factors associated with pregnancy outcomes in newborns from the czech republic Environ. Health Perspect 119 265–71 20923744 10.1289/ehp.1002470 PMC3040616

[57] Saenen ND 2016 Placental nitrosative stress and exposure to ambient air pollution during gestation: a population study Am. J. Epidemiol 184 442–9 27601048 10.1093/aje/kww007

[58] Nachman RM 2016 Intrauterine inflammation and maternal exposure to ambient pm2.5 during preconception and specific periods of pregnancy: the boston birth cohort Environ. Health Perspect 124 1608–15 27120296 10.1289/EHP243 PMC5047781

[59] Wright E 2017 Maternal vascular malperfusion and adverse perinatal outcomes in low-risk nulliparous women Obstetrics & Gynecology 130 1112 29016509 10.1097/AOG.0000000000002264

[60] Vadillo-Ortega F 2014 Air pollution, inflammation and preterm birth: a potential mechanistic link Med. Hypotheses 82 219–24 24382337 10.1016/j.mehy.2013.11.042 PMC3928635

[61] Sominsky L 2023 Pre-pregnancy obesity is associated with greater systemic inflammation and increased risk of antenatal depression Brain Behav. Immun 113 189–202 37437818 10.1016/j.bbi.2023.07.005

[62] Li K, Yang C, Fan J, Li X, Gu C and Liu H 2022 Prepregnancy body mass index, gestational weight gain, and maternal prepartum inflammation in normal pregnancies: findings from a Chinese cohort BMC Pregnancy and Childbirth 22 531 35768766 10.1186/s12884-022-04849-y PMC9245225

[63] Bernhardt GV 2022 Markers of inflammation in obese pregnant women: adenosine deaminase and high sensitive C—reactive protein Eur. J. Obstet Gynecol Reprod. Biol. X 16 100167 36312323 10.1016/j.eurox.2022.100167 PMC9597103

[64] Alcala M, Gutierrez-Vega S, Castro E, Guzman-Gutiérrez E, Ramos-Álvarez MP and Viana M 2018 Antioxidants and oxidative stress: focus in obese pregnancies Front Physiol. 9 1569 30459642 10.3389/fphys.2018.01569 PMC6232303

[65] Alderete TL 2017 Longitudinal associations between ambient air pollution with insulin sensitivity, β-cell function, and adiposity in los angeles latino children Diabetes 66 1789–96 28137791 10.2337/db16-1416 PMC5482082

[66] Kim JS 2018 Longitudinal associations of in utero and early life near-roadway air pollution with trajectories of childhood body mass index Environ. Health 17 64 30213262 10.1186/s12940-018-0409-7 PMC6137930

[67] McConnell R, Gilliland FD, Goran M, Allayee H, Hricko A and Mittelman S 2016 Does near-roadway air pollution contribute to childhood obesity? Pediatr Obes 11 1–3 25820202 10.1111/ijpo.12016 PMC4821543

[68] El Sherbiny S 2023 The effect of dietary patterns and nutrient intake on oxidative stress levels in pregnant women: a systematic review Antioxidants 12 1427 37507965 10.3390/antiox12071427 PMC10376333

[69] Hwang J, Shin D, Kim H and Kwon O 2022 Association of maternal dietary patterns during pregnancy with small-for-gestational-age infants: Korean mothers and children’s environmental health (MOCEH) study The American Journal of Clinical Nutrition 115 471–81 34625785 10.1093/ajcn/nqab340

[70] Aubert AM 2022 Predictors of maternal dietary quality and dietary inflammation during pregnancy: an individual participant data meta-analysis of seven European cohorts from the ALPHABET consortium Clinical Nutrition 41 1991–2002 35964423 10.1016/j.clnu.2022.06.042

[71] Haapala EA 2022 Associations of physical activity, sedentary time, and diet quality with biomarkers of inflammation in children European Journal of Sport Science 22 906–15 33599556 10.1080/17461391.2021.1892830

[72] Calcaterra V 2022 Use of physical activity and exercise to reduce inflammation in children and adolescents with obesity Int. J. Environ Res. Public Health 19 6908 35682490 10.3390/ijerph19116908 PMC9180584

[73] Llorente-Cantarero FJ 2021 Relationship between physical activity, oxidative stress, and total plasma antioxidant capacity in spanish children from the GENOBOX study Antioxidants 10 320 33672676 10.3390/antiox10020320 PMC7924393

